# Relationship between Socio-Demographic Factors and Adherence to Social Distancing Recommendations During Covid-19 Pandemic in Gonabad, Iran: A Cross-Sectional Study

**DOI:** 10.30476/IJCBNM.2021.90930.1747

**Published:** 2022-04

**Authors:** Fatemeh Hadizadeh-Talasaz, Ali Delshad Noghabi, Fatemeh Mohammadzadeh

**Affiliations:** 1 Department of Midwifery, School of Medicine, Social Development & Health Promotion Research Center, Gonabad University of Medical Sciences, Gonabad, Iran; 2 Social Development and Health Promotion Research Center, Gonabad University of Medical Sciences, Gonabad, Iran; 3 Department of Epidemiology and Biostatistics, School of Health, Social Development and Health Promotion Research Center, Gonabad University of Medical Sciences, Gonabad, Iran

**Keywords:** Adherence, COVID-19, Social distancing, Socio-demographic factors

## Abstract

**Background:**

Adherence to social distancing recommendations provided by health authorities plays an important role in the prevention and control of COVID-19 transmission, regarding the lack of effective drug therapies. Therefore, this study aimed to identify the relationship between socio-demographic factors and adherence to social distancing recommendations during COVID-19 pandemic in Gonabad, Iran.

**Methods:**

In this cross-sectional study, 587 subjects aged 15 and older were selected using the convenience sampling method from urban and rural areas in Gonabad, Iran, from July to September 2020. Data were collected online using a socio-demographic characteristics form, the adherence to social distancing recommendations scale, and a coronavirus disease awareness survey. Subsequently, the obtained data were analyzed in SPSS software (version 16) using hierarchical linear regression. The statistical significance was considered as P<0.05.

**Results:**

72.23% (95% confidence interval: 68.41%-75.82%) of the participants followed the recommendations of social distancing at the high level. Nevertheless, many differences were observed in the subgroups which resulted from the socio-demographic characteristics of the participants. According to the multiple linear regression model, sex (β=0.24, P<0.001) was the most important determinant of adherence to social behavioral recommendations, followed by occupation (β=0.15, P<0.001), age (β=0.14, P=0.007), awareness (β=0.13, P=0.001), and history of mental illness (β=-0.08, P=0.049).

**Conclusion:**

For the effective management of pandemics, the general public health policies should also consider the variability in responses and behavioral changes caused by socio-demographic characteristics, rather than focusing only on general measures, such as social distancing.

## INTRODUCTION

Coronavirus disease 2019 (COVID-19) is an acute respiratory infectious disease caused by severe acute respiratory syndrome coronavirus 2 (SARS-CoV-2). ^1^ This virus belongs to the SARS-like virus cluster. ^2^ The disease is highly contagious and spreads mainly through person-to-person contact and has various symptoms such as pneumonia, fever, breathing difficulty, and lung infection. ^1 , 3^ COVID-19 pandemic, as a global public health crisis, is a serious threat to humanity and has health, social, and economic consequences, including interruption in education and schooling, job loss, violence, and addiction. ^4 - 8^ Although the symptoms of COVID-19 are in most cases relatively mild and without serious outcomes, approximately 4% of the world’s population would be severely infected if they are exposed to COVID-19 and need to be hospitalized for treatment. ^9^ At the time of writing this manuscript (June 18, 2021), the total number of confirmed infected cases and deaths caused by this disease was estimated at 3,070,426 and 82,619 in Iran, respectively. ^10^

The enforcement of widespread holiday programs to reduce the transmission of infection had negative social and economic consequences in many countries; therefore, some steps should be taken to revive social and economic life. The most important step is to preserve public life while minimizing the risk of infection. ^9^ Most governments implemented a variety of non-pharmacological strategies through changing social and health behaviors in order to prevent COVID-19 transmission and control the epidemic. ^5 , 11^ In addition to the observation of personal hygiene, such as hand washing and wearing masks, the reduction of interactions between people through social distancing is an effective and common public health measure to reduce the risk of infection and the spread of the virus in the community. ^9^

Social distancing, which is defined as maintaining a distance of at least 3-6 feet (1-2 meters) from other people who are not in the same home, is a key behavior in minimizing COVID-19 infection. ^1^ Due to the lack of known drug therapies, the course of the COVID-19 epidemic depends to a large extent on how the population complies with the rules and adheres to the behavioral recommendations provided by health authorities, including social distancing. ^5^ Therefore, adherence to the recommendations of social distancing, such as not traveling, limiting the physical contact with people outside home, and observing 1-2m distance from others when in public, is the main strategy in the prevention and control of COVID-19 transmission. ^5 , 12^

Based on the results of a previously conducted study, researchers have concluded that a set of social distancing measures may have an impact on the potential control of influenza pandemic in the future. ^13^ Therefore, it seems reasonable to consider social distancing interventions as an alternative or complement to antiviral-based strategies. ^13^ According to some studies, small changes in behavior can have major impacts on transmission patterns during an epidemic. Some researchers have argued that social distancing, if started quickly, can stop an epidemic for a relatively long period of time. ^13 , 14^ With the outbreak of COVID-19, officials in Iran, like those in other countries, implemented social distancing recommendations for the closure of schools and universities, mosques, shrines, Friday prayers, government jobs (except for essential jobs), and cancellation of sport events. ^15^ However, these recommendations were not followed by all people.

Given the fact that social distancing measures make a difference in people’s lives and may potentially be needed for the months or years to come, it is important to understand what facilitates or prevents adherence to these measures. In other words, behavior change is the only way for preventing the spread of COVID-19, and understanding behaviors is a key to changing them. ^16^

Little is known about the factors influencing compliance with social distancing recommendations during pandemics. In the context of the COVID-19 pandemic, individual motives or reasons seem to play a role in adherence to social distancing recommendations (e.g. a desire to protect oneself and others). According to the results of some conducted studies, motivations may be related to various socio-demographic variables. ^8^

Preventive behaviors have a significant relationship with some socio-demographic characteristics. Self-care, which is the activities and precautions that a person takes to prevent or control infections, is influenced by factors such as age, lifestyle, health status, emotional state, and knowledge. ^17^

The results of a study in Japan (2021) showed that socio-demographic and personal characteristics of individuals played an important role in shaping the prevention measures of COVID-19 and thus in controlling the spread of pandemics. ^18^

Social distancing is an important health care behavior that is influenced by various factors, including socio-demographic characteristics of individuals, which can vary in different countries and societies. Identification of determinants of adherence to social distancing recommendations during and following a pandemic may provide essential evidence for more successful and appropriate management of the pandemic and behavioral interventions. Since there is little information about the relationship between the above-mentioned variables in Iran, this study aimed to identify the relationship between socio-demographic factors and adherence to social distancing recommendations during COVID-19 pandemic in Gonabad, Iran.

## METHODS

This cross-sectional study was conducted from July 7, 2020 to September 20, 2020, in Gonabad, Iran, when strict regulations on social distancing were implemented in the country. This city is more exposed to COVID-19 due to its elderly population. ^19^ The inclusion criteria in this study included the age of 15 years old and over, the ability to read and write in Persian, internet access, and residence in Gonabad, Iran. Exclusion criteria was unwillingness to continue participation while responding. To determine the status (mean score) of social distancing in COVID-19 pandemic in Gonabad, we calculated the minimum sample size at 546 cases based on the formula with a confidence interval of 95%, a test power of 0.8, and a small effect size E=0.12, according to Cohen’s guidelines, ^20^ which was increased to 600 individuals considering a 10% attrition rate. Questionnaires could only be submitted if they were complete. Therefore, there was no case with missing data.

The sample size was large enough and supported the rule of thumb (n≥50+8 m; m=the number of predictors) for regression analysis. ^21^ The study protocol was approved by the Ethics Committee of Gonabad University of Medical Sciences, Gonabad, Iran (IR.GMU.REC.1399.052). No identifiable information (e.g. name, contact information) was collected from the participants. The return of completed questionnaires was regarded as the informed consent of the participants.

The subjects were selected through the convenience non-probability sampling technique. The data were collected through online questionnaires. We sent a link of the web page of the electronic questionnaires to the participants via the available social networks (e.g. Telegram and WhatsApp groups) and invited them to participate in the study. The online questionnaire has the advantage to reach a large audience. Participants could enter the pages of the questionnaire by studying and understanding the objectives of the study (mentioned at the beginning of the questionnaire) and clicking the “I agree” option, in case they agreed to participate in the study. Participation in the study was voluntary and the participants could withdraw from the study any time they wanted. It is worth mentioning that only questionnaires without missing answers could be sent.

Data collection tools included socio-demographic characteristics questionnaire, coronavirus awareness questionnaire, and adherence to social distancing recommendations scale. Socio-demographic characteristics questionnaire included a wide range of items, such as age, sex, marital status, place of residence, occupation, level of education, family income level, and social class (subjective). The participants were also asked about their history of chronic illness; mental illness; history of infection or death due to COVID-19 in themselves or the people around including family, neighbors, or friends; smoking; and drug use.

 Coronavirus awareness questionnaire was a researcher-made questionnaire consisting of 20 three-choice (true, false, and I do not know) items that were scored zero and one. Each “false” and “I do not know” response was scored zero, and each correct answer was scored one. The total score was obtained by summing the scores which ranged from 0 to 20. Higher scores indicate greater awareness of coronavirus disease. 

Adherence to social distancing recommendations scale consisted of a list of 22 behaviors in four sections including interaction with people, self-care measures, social and daily activities, and travel. The behavioral list was developed by the research team, in line with the social distancing recommendations of health officials. The participants’ adherence to each behavior over the past month was scored on a 5-point Likert scale of 1=Never, 2=Rarely, 3=Sometimes, 4=Often, and 5=Always. The final score was the sum of the scores of each section; therefore, the minimum and the maximum scores were 22 and 110, respectively. The mean score in this questionnaire was obtained by the sum of scores divided by the number of the statements. The obtained score indicated the extent of adherence to the social distancing recommendations. A higher score indicates more adherence. Based on the quartiles of the distribution of the scores, the mean scores of adherence were also categorized as none/very low, low, moderate, and high.

The validity of the questionnaires was assessed using content and face validity. A panel of experts assessed the validity of the content of the coronavirus awareness questionnaire and adherence to social distancing recommendations scale using both qualitative and quantitative methods. In the qualitative method, the prepared questionnaire was sent to 10 faculty members, and their opinions about the questionnaires were gathered. Furthermore, content validity ratio (CVR) and content validity index (CVI) were utilized in the quantitative method.

The CVR was assessed by 10 experts who reviewed each item based on a three-part spectrum (3= necessary, 2=useful but not necessary, and 1=not necessary). The items with a CVR numerical value higher than 0.62 (based on the evaluation of 10 experts) were preserved based on the *Lawshe* table of determining the minimum value. ^22 , 23^ At this stage, by removing two items in the coronavirus awareness questionnaire and one item in the adherence to social distancing recommendations scale, the number of items was reduced to 20 and 22, respectively. The CVI was also calculated for the remaining items in the tool. For this purpose, the criteria of relevancy was examined by 10 experts for each item on a 4-point Likert scale. Two forms of CVI containing I-CVI and S-CVI (S-CVI/Ave and S-CVI/UA) were evaluated. In all items, I-CVI was greater than 0.8. S-CVI/Ave and S-CVI/UA for the coronavirus awareness questionnaire were 0.90 and 0.81 and for the adherence to social distancing recommendations scale 0.85 and 0.78, respectively. To evaluate the reliability, the questionnaires were completed by 20 participants, and the reliability of the coronavirus awareness questionnaire and adherence to social distancing recommendations scale were confirmed by the Cronbach’s alpha coefficient of 0.86 and 0.82, respectively.

 All statistical analyzes were performed using SPSS software (version 16). Quantitative variables were reported as mean±standard deviation (SD), and qualitative variables were reported as number and percentage. The default normality of quantitative variables was examined using the Kolmogorov-Smirnov test. The linear regression model was adopted to determine the factors related to the adherence to the social distancing recommendations. Therefore, the variables that had P<0.15 in simple linear regression were entered into the multiple linear regression model, and their relationship was assessed in the presence of other variables following the social distancing recommendations. A p-value less than 0.05 was considered statistically significant. 

The assumptions of the linear regression model, including normality, variance homogeneity, and independence of the residuals were examined using Kolmogorov-Smirnov tests, plots of standardized residuals versus predicted values, and residual time series plot, respectively. Multicollinearity assumption was also checked using variance inflammation factor (VIF).

## RESULTS

Out of 600 participants, 13 failed to meet all the inclusion criteria; therefore, data of 587 individuals were analyzed. The mean age of the participants was estimated at 35.37±11.66 years (age range: 15-80 years). Other characteristics of the participants are listed in Table 1.

**Table 1:** Characteristics of the study participants

Characteristics	N (%)
Sex	
Male	122 (20.78)
Female	465 (79.22)
Marital status	
Married	410 (69.85)
Single/widowed/divorced	177 (30.15)
Place of living	
City	521 (88.76)
Village	66 (11.24)
Job					
Employee	243 (41.40)
Student	122 (20.78)
Housewife	115 (19.60)
Retired	37 (6.30)
Unemployed	13 (2.21)
Other	57 (9.71)
Educational level		
High school or below	162 (27.60)
Associate or Bachelor degree	311 (52.98)
Master degree or higher	114 (19.42)
Family income level		
Low	103 (17.55)
Moderate	432 (73.59)
High	52 (8.86)
Social class (subjective)		
Low	96 (16.35)
Middle	434 (73.94)
High	57 (9.71)
Smoking/Hookah	
Yes	34 (5.79)
No	553 (94.21)
Drug abuse	
Yes	6 (1.02)
No	581 (98.98)
History of chronic diseases	
Yes	81 (13.80)
No	506 (86.20)
History of mental disorders	
Yes	15 (2.56)
No	572 (97.44)
History of COVID-19 infection	
Yes	31 (5.28)
No	556 (94.72)
History of COVID-19 infection for people around	
Yes	300 (51.11)
No	287 (48.89)
History of death due to COVID-19 for people around	
Yes	66 (11.24)
No	521 (88.76)
Information sources about COVID-19*				
TV & Radio	214 (36.46)
Internet	278 (47.36)
Health staff	87 (14.82)
Friends/relatives/neighbors	17 (2.90)

*Each individual could use several information sources

The mean score of total awareness about COVID-19 was determined at 17.98±1.85, and the rates of correct answers to the questions were estimated between 60.13% and 98.97%. 

Adherence to social distancing recommendations was at a moderate to high level in most behaviors, so that based on the quartiles of the scores distribution, the adherence of 362 subjects (61.67%) was at a moderate and 225 people (38.33%) at a high level. The mean total score and in each of the subscales of adherence to the recommendations of social distancing and the extent to which individuals adhered to each of the items of social distancing behaviors are shown in Table 2.

**Table 2:** Frequency distribution of adherence to the recommendations of social distancing during the COVID-19 pandemic

Preventive behaviors	Mean±SD (Min, Max)	Never/rarely	Sometimes	Often	Always
		N (%)	N (%)	N (%)	N (%)
Interaction with people	4.22±0.53 (1.67, 5.00)				
1. Limiting face-to-face interaction with people		11 (1.87)	67 (11.41)	339 (57.76)	170 (28.96)
2. Canceling or postponing unnecessary appointments		7 (1.20)	50 (8.52)	245 (41.73)	285 (48.55)
3. Interruption or reduction of family socializations		7 (1.20)	69 (11.75)	333 (56.73)	178 (30.32)
Self-care measures	4.54±0.33 (3.11, 5.00)				
4. Staying at home		13 (2.21)	73 (12.44)	391 (66.61)	110 (18.74)
5. Avoid going to crowded places		8 (1.36)	31 (5.28)	229 (39.01)	319 (54.35)
6. Avoiding attendance in public places (such as restaurants, gyms, mosques, parks, weddings, mourning sessions, etc.)		10 (1.71)	24 (4.09)	122 (20.78)	431 (73.42)
7. Observing a distance of 2 meters with others in case of compulsion to attend a social environment		6 (1.02)	70 (11.93)	282 (48.04)	229 (39.01)
8. Reducing social interactions in the workplace if forced to attend work		1 (0.17)	22 (3.74)	127 (21.64)	437 (74.45)
9. Using a face mask if forced to attend a social setting		7 (1.20)	58 (9.88)	261 (44.46)	261 (44.46)
10. Avoiding kissing others		2 (0.34)	6 (1.02)	28 (4.77)	551 (93.87)
11. Avoiding shaking hands with others		3 (0.51)	9 (1.53)	50 (8.51)	525 (97.95)
12. Avoiding contact with people with colds or those with flu-like symptoms		1 (0.17)	21 (3.58)	106 (18.05)	459 (78.20)
Performing social and daily activities	4.21±0.44 (2.50, 5.00)				
13. Reducing going to school/university/workplace		42 (7.16)	87 (14.82)	207 (35.26)	251 (42.76)
14. Doing things remotely or online if possible		39 (6.64)	80 (13.63)	235 (40.04)	233 (39.69)
15. Canceling or postponing meetings with friends as well as sports programs		4 (0.68)	37 (6.30)	158 (26.92)	388 (66.10)
16. Reducing store visits		3 (0.51)	72 (12.27)	370 (63.03)	142 (24.19)
17. Daily shopping by phone and receiving orders at home		131 (22.32)	115 (19.59)	216 (36.80)	125 (21.29)
18. Shopping and visiting stores at less crowded times if needed		14 (2.39)	93 (15.84)	283 (48.21)	197 (33.56)
19. Reducing the use of public transport		22 (3.74)	35 (5.96)	116 (19.76)	414 (70.54)
20. Using of personal vehicle		6 (1.02)	18 (3.06)	103 (17.56)	460 (78.36)
Taking trips	4.78±0.43 (1.50, 5.00)				
21. Avoiding unnecessary trips		2 (0.34)	7 (1.20)	85 (14.48)	493 (83.98)
22. Reducing the number of necessary trips		3 (0.51)	15 (2.56)	113 (19.25)	456 (77.68)
Adherence to the recommendations of social distancing (total)	4.40±0.32 (3.09, 5.00)

The results of the study showed that most of the participants always followed some social distancing behaviors, including avoiding attendance in public places such as restaurants, gyms, cinemas, mosques, parks, weddings, mourning sessions, etc. (73.42%, 95% CI:69.65%-76.95%), reducing social interactions in the workplace if forced to attend work (74.45%, 95% CI:70.71%-77.92%), avoiding kissing others (93.87%, 95% CI: 91.61%-95.66%), avoiding shaking hands with others (97.95%, 95% CI: 96.45 %-98.93%), avoiding contact with people with colds or those with flu-like symptoms (78.20%, 95% CI:74.63%-81.47 %), reducing the use of public transport (70.54%, 95% CI:66.65%-74.19%), using personal vehicles (78.36%, 95% CI: 74.80%-81.63%), avoiding unnecessary trips (83.98%, 95% CI :80.76%-86.86%), and reducing the number of necessary trips (77.68%, 95% CI: 74.09%-80.99%). Among social distancing behaviors, daily shopping by phone and receiving orders at home was less reported (21.29%, 95% CI: 18.04%-24.83%), and the participants did this behavior sometimes and rarely/not at all (Table 2).

Table 3 displays the results of linear regression analysis to examine the relationship between socio- demographic factors and adherence to the recommendations of social distancing. Based on the simple linear regression model, age, sex, occupation, smoking or hookah use, history of mental illness, and awareness score related to adherence to the social distancing recommendations had a P value<0.15 and were entered into a multiple linear regression model. Based on the values of standardized regression coefficients (β) in the multiple linear regression model, sex was the most important determinant of adherence to behavioral recommendations (β=0.24), followed by occupation (β=0.15), age (β=0.14), awareness. (β=0.13), and a history of mental illness (β=-0.08). The mean score of adherence to behavioral recommendations was significantly 0.19 units higher in females than males (B=0.19, P<0.001). Moreover, the mean score of adherence to behavioral recommendations in housewives was 0.13 units higher, compared to other occupations, which was statistically significant (B=0.13, P<0.001). Older people reported a higher score of adherence to behavioral recommendations, so that per each year increase in the age score, the behavior score increased by 0.004 units (B=0.004, P=0.007). In addition, each unit increase in awareness score was associated with 0.02 units increase in behavior score (B=0.02, P=0.001). Furthermore, in people with a history of mental illness, the mean score of adherence to the behavioral recommendations was 0.15 units lower than that in those who did not have a history of mental illness (B=-0.15, P=0.049) (Table 3).

**Table 3:** Results of linear regression model on the factors associated with adherence to the recommendations of social distancing

Predictors	Simple regression					Multiple regression				
	B	Beta	S.E.	t	P-value	B	Beta	S.E.	t	P-value
Age	0.01	0.09	0.001	2.29	0.02*	0.004	0.14	0.001	2.69	0.007
Sex										
Female a	0.23	0.28	0.03	7.17	<0.001*	0.19	0.24	0.03	5.59	<0.001
Marital status										
Married b	0.03	0.04	0.02	0.87	0.39	--	--	--	--	--
Place of living										
City c	0.02	0.02	0.04	0.45	0.65	--	--	--	--	--
Job d				
Student	0.02	-0.02	0.03	-0.63	0.53	0.02	0.03	0.04	0.54	0.54
Housewife	0.16	0.19	0.03	4.47	<0.001*	0.13	0.15	0.04	3.58	<0.001
Retired	0.07	0.06	0.06	1.37	0.17	0.04	0.03	0.06	0.73	0.47
Unemployed	0.11	0.05	0.09	1.26	0.21	0.12	0.05	0.09	1.31	0.19
Other	0.13	-0.12	0.05	-2.83	0.21	-0.08	-0.07	0.05	-1.70	0.09
Educational level										
College e	0.02	0.03	0.03	0.61	0.54	--	--	--	--	--
Family income level f										
Moderate	0.04	0.05	0.05	0.85	0.39	0.02	0.03	0.04	0.57	0.57
High	0.10	0.12	0.06	1.80	0.07*	0.09	0.11	0.05	1.76	0.08
Social class f										
Middle	0.03	0.04	0.03	0.74	0.56	--	--	--	--	--
High	0.05	0.05	0.05	0.92	0.79	--	--	--	--	--
Smoking/Hookah
Yes g	0.13	-0.09	0.06	2.30	0.02*	0.02	0.02	0.06	0.46	0.64
Drug abuse										
Yes g	0.04	-0.01	0.13	0.30	0.77	--	--	--	--	--
History of chronic diseases
Yes g	0.03	-0.03	0.04	-0.83	0.41	--	--	--	--	--
History of mental disorders										
Yes g	0.12	-0.06	0.08	-1.53	0.13*	-0.15	-0.08	0.08	-1.97	0.049
History of COVID-19 infection:
Yes g	0.01	0.01	0.06	0.11	0.913	--	--	--	--	--
History of COVID-19 infection for people around										
Yes g	0.03	0.05	0.03	1.27	0.20	--	--	--	--	--
History of death due to COVID-19 for people around	
Yes g	0.01	-0.01	0.01	-0.04	0.97	--	--	--	--	--
Knowledge score	0.03	0.15	0.007	3.63	<0.001*	0.02	0.13	0.006	3.38	0.001

S.E., Standard Error;

a reference category=Male;

b reference category=Single/widowed/divorced;

c reference category=Village;

d reference category=Employee;

e reference category=High school or less;

f reference category=Low;

g reference category=No; *P<0.15

## DISCUSSION

This study aimed to determine the relationship between socio-demographic factors and adherence to social distancing recommendations during COVID-19 pandemic in Gonabad, Iran. The results showed that adherence to social distancing recommendations was relatively high in most behaviors. Despite the high rate of adherence to social distancing recommendations in the whole population, large differences were observed in the behavioral response to COVID-19 in terms of socio-demographic characteristics of individuals. According to the multiple linear regression model, sex was the most important determinant of adherence to behavioral recommendations, followed by occupation, age, awareness, and history of mental illness.

Based on the obtained results, adherence to the preventive behavior of social distancing was higher in women than men. The sex difference in this study was in line with that in previous studies in which women were more cautious than their male counterparts during the COVID-19, ^12 , 24 - 27^ as well as the swine flu pandemics. ^28^ In addition, they refrained from social activities and kept a safe distance in public places. ^12^

A meta-analysis showed that female respondents had 50% more protective behaviors in response to respiratory disease epidemic and pandemic, compared to their male counterparts. ^29^ This sex gap was also observed in the COVID-19 pandemic. In one study, being a woman was associated with staying more days at home and greater adherence to social distancing behaviors during the COVID-19 pandemic. ^26^

According to the findings of previous studies, women were more protective against infections than men during previous epidemics. ^30^ Therefore, sex differences played a role in how people perceived and responded to health-related risks, regardless of their age, level of education, or even employment status. ^5^ According to the studies, this difference can be attributed to the fact that women are more likely than men to report a higher level of risk as a concern. Other possible reasons include social roles. Women avoid risks more than men due to their role as nurturers and caregivers. However, men are less likely than women to avoid risks as a result of their role as income-generators. ^25^ Therefore, based on the results of the mentioned studies, sex-specific programs should be implemented to strengthen preventive behaviors during COVID-19.

Moreover, the results of the present study revealed that adherence to social distancing recommendations was more common in housewives than in other occupations. The results of some studies indicated that there was a positive and significant relationship between the status of occupation and adherence to social distancing behavior, so that the observance of social distancing was higher among non-regular employees. In contrast, employees with regular jobs considered it difficult to adhere to social distancing behaviors. ^18^ In another study, people’s work status had an impact on their adherence to protective measures during a pandemic. ^30^ Therefore, the findings of this study confirm that occupation status is a factor that should be considered in managing the pandemic. According to the results of the present study, protective behaviors, including social distancing, was observed more in older individuals. One reason for the observation of greater protection in older people is that they are more exposed to the underlying diseases. The findings of this study are in line with the results of previous studies ^26^ including a study conducted in Europe and North America (2020). They reported that older people (>45 years) refused to participate in social activities more than young people (18-24 years) during COVID-19 and kept a safe distance in public places. ^12^

The need to receive social support or a sense of belonging in younger people might be one of the reasons for their lower adherence to social distancing behaviors. ^12^ The results of other studies have indicated that some socio-demographic characteristics (sex, age) were associated with adherence to public health recommendations during COVID-19. ^31 , 32^ According to the results of another study, women and the elderly feel more vulnerable and have a greater sense of responsibility and the desire to protect the society. ^33^

The results of this study are not in the same line with another study reporting that young participants were more likely to stay at home due to having more entertainment options. ^25^ According to the results of the present study, young people are more vulnerable to COVID-19 infection. The reason, according to a similar study, is that they observe social distancing less commonly. ^27^ Therefore, it is necessary to plan to educate young people and encourage them to engage in more protective behaviors.

Another result of the present study is that increasing awareness has increased the probability of adherence to the recommendations of social distancing. The results of this study were in line with those of a study conducted in China in which knowledge had a significant positive impact on health behavior against COVID-19. ^31^ Based on the results of a review study, public awareness of the disease and the quarantine methods was one of the important factors influencing decisions to adhere to quarantine during the outbreak of infectious diseases. ^34^

Based on the results of the present study, in people with a history of mental illness, adherence to the behavioral recommendations of social distancing was lower. According to a study in Japan, people with anxiety and depression are significantly less likely to engage in a variety of preventative behaviors, including washing their hands, wearing a mask, avoiding crowds, and staying at home. ^35^ A study in China also reported that depressive symptoms might inhibit preventive behaviors in response to pandemic diseases such as COVID-19. ^36^ Therefore, for preventing the spread of the epidemic, the importance of counseling during an epidemic in people with mental health problems is emphasized. ^35^

The attempts to reduce the speed of virus transmission during epidemics require significant behavioral changes. ^37^ Not only do behaviors reduce the risk of infection in the individual, but they also limit the spread of the disease to others. ^8^

Governments tend to implement some standard public measures during public health emergencies and expect everyone to follow the recommendations. ^18^ This happened at the beginning of the COVID-19 outbreak in Iran and many other countries. Individual’s beliefs are different, and they are influenced by socio-demographic characteristics that may affect their adherence to public action; therefore, governments should focus on identifying vulnerable populations that tend to avoid adherence to the recommendations, rather than focusing on the public measures. ^18^

Participants’ self-report, non-coverage of all age groups, and online sampling due to epidemic conditions were among the limitations of this study. Despite the limitations, this study provided useful information about the relationship between socio-demographic characteristics of individuals and behavioral changes and the need to pay attention to these characteristics for effective management of pandemic diseases. 

## CONCLUSION

For more effective management of a pandemic, public health policies should also consider variability in responses and behavioral changes due to socio-demographic characteristics instead of focusing only on public actions. It seems that the implementation of educational and health programs can encourage people to follow social distancing recommendations and change the individuals’ behaviors by targeting specific demographic groups and focusing on individuality, which in turn could lead to the mitigation of the COVID-19 outbreak. 

In this study, we only examined the rrelationship between socio-demographic characteristics of individuals and adherence to social distancing recommendations. It is suggested that more researches be done on the effect of personality and emotional characteristics of individuals on adherence to social distancing.

## ACKNOWLEDGEMENT

This study was approved (approval No: A-10-1660-6) by the Social Development & Health Promotion Research Center, Gonabad University of Medical Sciences, Gonabad, Iran. The authors acknowledge Social Development & Health Promotion Research Center for its support and all the people who participated in the study.

**Conflict of Interest:** None is declared. 
